# The choroid plexus is a source of active renin in the central nervous system

**DOI:** 10.21203/rs.3.rs-10474429/v1

**Published:** 2026-07-30

**Authors:** Ismary Blanco, Haley Masters, Samantha Reasonover, Dandan Liu, Panpan Zhang, Yukti Vyas, David A. Davis, Mingzhi Zhang, Raymond C. Harris, Timothy J. Hohman, Neil Dani, Ethan S. Lippmann, Angela L. Jefferson, Monica M. Santisteban

**Affiliations:** 1 Department of Medicine, Division of Genetic Medicine and Clinical Pharmacology, Vanderbilt University Medical Center, Nashville, TN; 2 Department of Biomedical Engineering, Vanderbilt University, Nashville, TN; 3 Vanderbilt Memory and Alzheimer’s Center, Vanderbilt University School of Medicine, Nashville, TN; 4 Vanderbit Alzheimer’s Disease Research Center, Nashville, TN; 5 Department of Biostatistics, Vanderbilt University Medical Center, Nashville, TN; 6 Department of Neurology, Brain Endowment Bank, University of Miami Miller School of Medicine, Miami, Florida, 33136, USA; 7 Department of Medicine, Division of Nephrology and Hypertension, Vanderbilt University Medical Center, Nashville, TN; 8 Department of Neurology, Vanderbilt University Medical Center, Nashville, TN; 9 Department of Cell and Developmental Biology, Vanderbilt University, Nashville, TN

## Abstract

The renin–angiotensin system is important in cardiovascular and brain physiology. The local brain RAS was proposed over 50 years and decades of research support its existence. However, it remains controversial primarily due to disputed identification of functional renin in the central nervous system. Here, we show that functional renin is produced and released by the choroid plexus, the primary source of cerebrospinal fluid in the brain. Across public RNA sequencing datasets, renin is expressed in both mouse and human choroid plexus epithelial cells, and its expression is validated by RNAscope, immunofluorescence, qPCR, and western blotting. We further demonstrate renin activity in human cerebrospinal fluid that is independent of either circulating renin or blood–brain barrier integrity and confirm its release and activity from mouse choroid plexus explants. Notably, cerebrospinal fluid renin activity but not plasma renin activity increases with aging in human adults. These findings identify the choroid plexus as a source of renin in the central nervous system and provide evidence supporting a local functional brain renin angiotensin system.

## Introduction

The renin–angiotensin system (RAS) is best known as an endocrine pathway that governs blood pressure and fluid balance, yet its influence extends beyond the periphery. Renin, the rate-limiting enzyme in the generation of angiotensin II (Ang II), was discovered in the 1890s and decades later reported to be functionally active in different tissues including the brain^1–5^. Foundational studies identified the role of the brain-specific renin isoform, renin-b, as an endogenous regulator of central RAS activity and cardiovascular homeostasis^5,6^. Despite these and numerous other studies indicating the presence and importance of the brain RAS^7–10^, its existence remains under debate, particularly due to a lack of consensus on the presence of functional renin in the brain^11^. Last year, a landmark paper by Fekete et al showed indisputable evidence of the presence of renin production in neurons in the nucleus ambiguus^12^. Together, these studies established the presence of RAS components within the central nervous system (CNS), while highlighting a major unresolved question: whether renin exists in sufficient quantities, and in the appropriate anatomical compartments, to support a truly local and widespread brain RAS. In this regard, the choroid plexus (ChP) is particularly intriguing because it produces the majority of CSF, acts as a secretory organ, and has long been reported to express high levels of renin.

The ChP is a highly vascularized epithelial structure located within the brain ventricles that plays a critical role in CNS homeostasis. As the primary source of CSF^13^ and the site of the blood–CSF barrier, the ChP regulates nutrient and waste exchange, immune cell trafficking, and CNS immune surveillance^14^. Beyond its barrier functions, the ChP is increasingly recognized as a secretory organ that releases metabolites, cytokines, growth factors, and extracellular vesicles into the CSF, enabling communication with brain regions throughout the CNS^15,16^. Consequently, molecules produced by the ChP, including renin, have the potential to influence brain function far beyond the ventricular system through CSF-mediated signaling. Therefore, in this study, we investigated whether the ChP serves as a functional source of renin and whether renin activity is present in human CSF independently of circulating renin activity.

## Results

### RNAseq studies show renin expression in ChP

**Figure 1.**
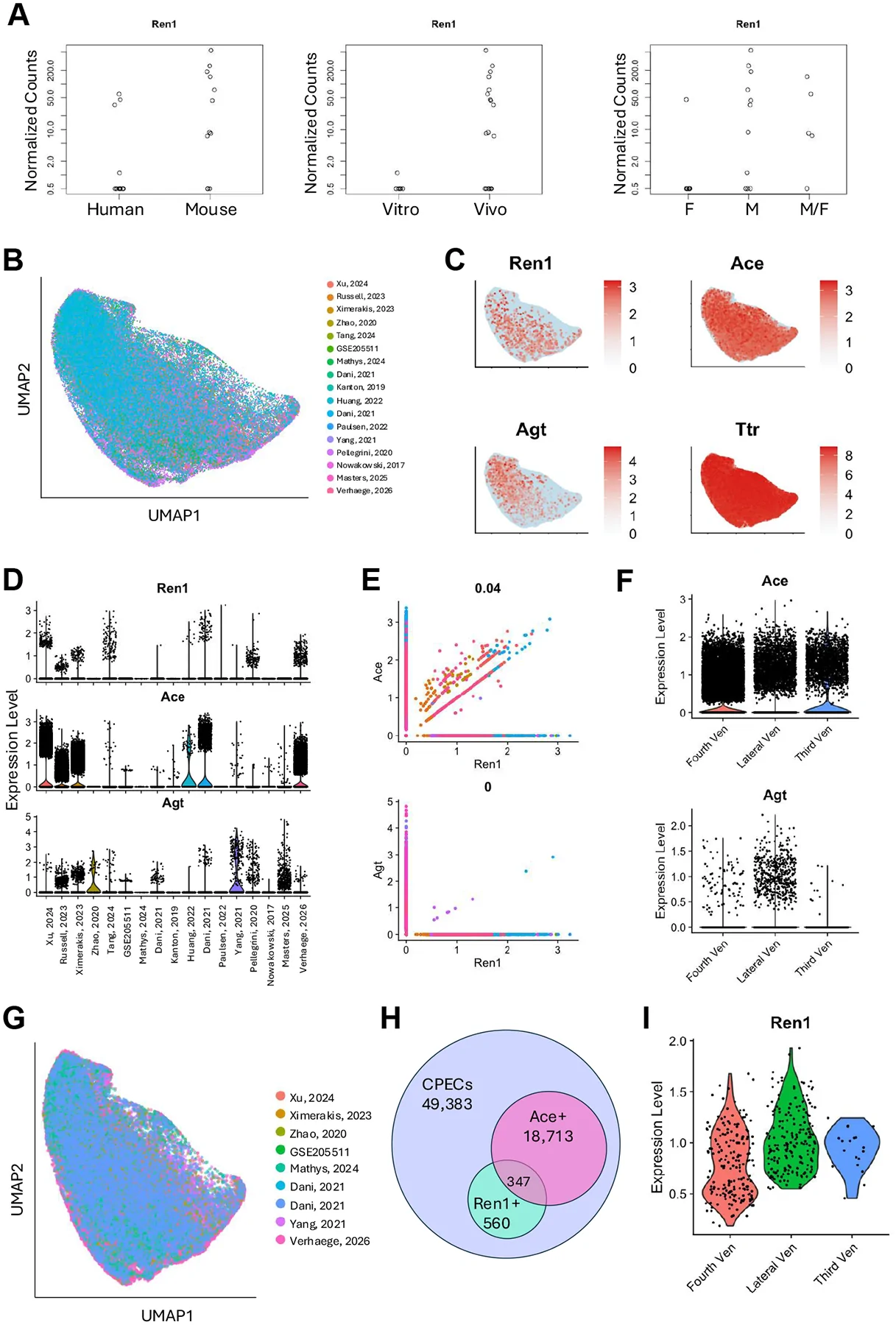
Choroid plexus epithelial cells express various RAS components. **a,** Pseudobulk analysis of renin expression between species (mice and humans) and sex (female and male), but higher expression *in vivo* than *in vitro*. **b,** Single-cell resolution datasets from mice and humans reveal **c,** expression of renin (*Ren1*), angiotensin converting enzyme (*Ace*), and angiotensinogen (*Agt*) in CPECs and **d,** across the different datasets. **e,** Co-express *Ace* and *Ren1* resulted in a correlation value of 0.04 whereas co-express *Agt* and *Ren1* resulted in a correlation value less than 0.001. **f,** *Ace* and *Agt* expression across 4^th^ ventricle (Ven), lateral ventricles, and 3^rd^ ventricle. **g,** Single-cell resolution datasets containing at least 3 Ren1+CPECs. **h,** Approximately 1% of CPECs express renin (total CPECs = 46699; *Ren1*+ cells = 440) and around 34% express *Ace* (15824). i, Comparison of renin expressing CPECs between the 4^th^, lateral, and 3^rd^ ventricles.

We assessed renin expression across publicly available RNA sequencing datasets containing mouse and human ChP epithelial cells (CPECs)^17–36^ (**Supp. Table 1**). Human datasets were converted to the mouse nomenclature for the gene name using Ensembl’s BioMart package^37^. Using a pseudobulk analysis of CPEC datasets with positive renin expression in at least 1 CPEC^17,19–21,23,24,27,31,36^, we found that renin RNA expression is higher *in vivo* than *in vitro*, comparable between male and female, and between mice and humans (Fig. 1A). *Ace* and *Agt* RNA expression were comparable between in vivo and in vitro, between mice and humans, and between male and female (**Supp. Fig. 1A-B**). *Ren1*, but not *Ace* or *Agt*, expression was also higher in adult vs young (**Supp. Fig. 1C**). Focusing on the datasets with single-cell resolution from both mice and humans^17,24,27–31,18–21,23,25,26,36^ (Fig. 1B), we mapped the cell expression of renin (*Ren1*), angiotensin converting enzyme (*Ace*), and angiotensinogen (*Agt*) both across cells (Fig. 1C) and across studies (Fig. 1D). We then assessed the overlap expression of *Ren1* with *Ace* and *Agt*, individually (Fig. 1E). Focusing on studies with ventricle location specification^24^, we found comparable expressions of *Ace* across hindbrain (4^th^ ventricle), telencephalic (lateral ventricles), and diencephalic (3^rd^ ventricle), with *Agt* having higher cell counts in the lateral ventricles (Fig. 1F). We then focused on single cell resolution studies^17,19–21,23,24,28,36^ and at least 3 CPECs expressing *Ren1* to account for false negatives due to sequencing depth (Fig. 1G). We find that approximately 1.13% of CPECs express renin and about 60% of these co-express *Ace* (Fig. 1H). Finally, we filtered *Ren1* expressing CPECs in datasets with ventricular specification, and observed that *Ren1* expression appeared slightly higher in the lateral ventricles compared to the fourth ventricle (Fig. 1I). These results provide evidence from multiple independent groups that mouse and human ChP epithelial cells express renin mRNA.

### Renin is expressed by choroid plexus epithelial cells in mice and humans

**Figure 2.**
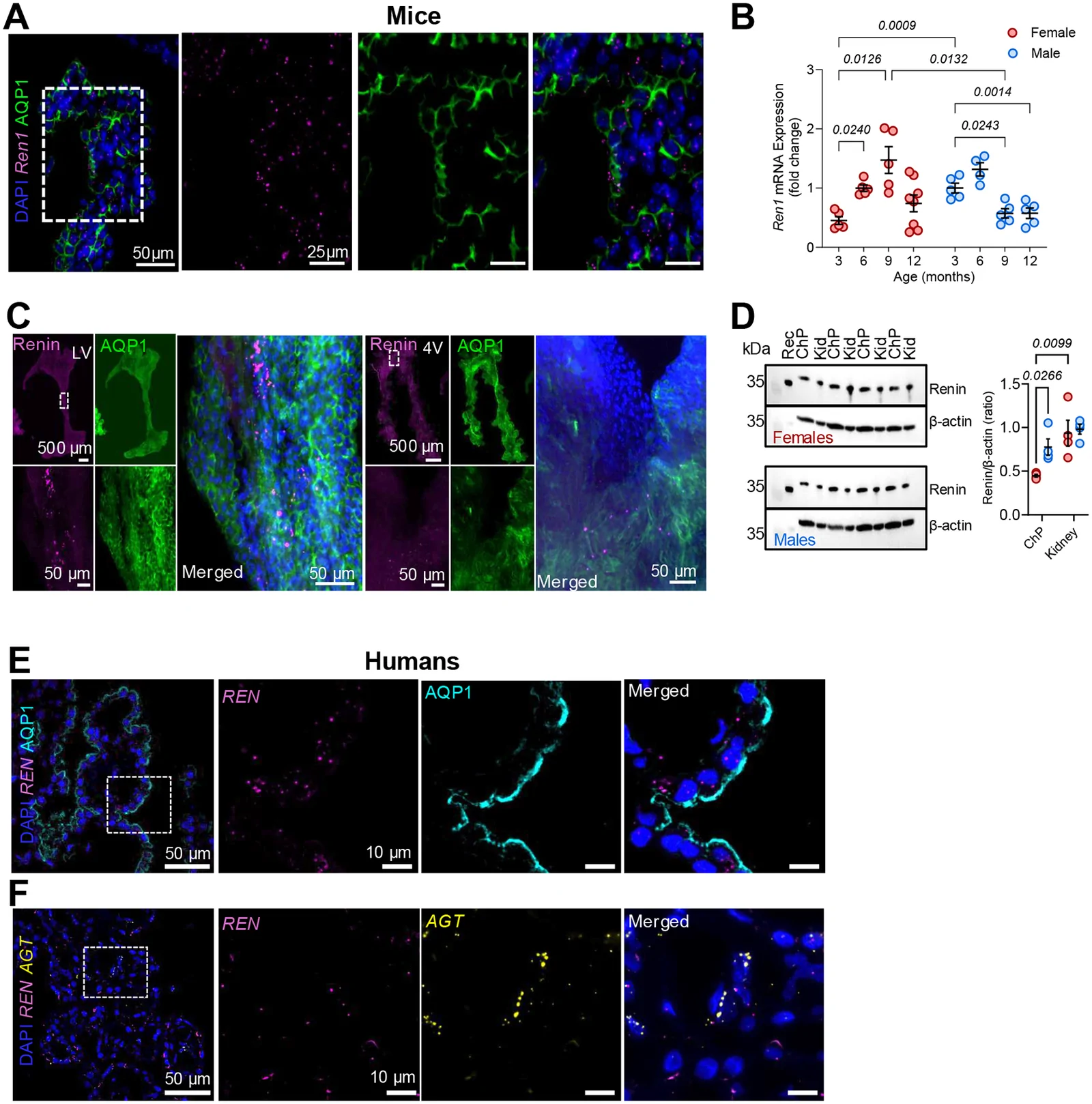
Renin expression in mouse and human choroid plexus epithelial cells. **a,** Representative coronal images of mice choroid plexus tissue showing Ren1 mRNA (magenta), aquaporin 1 (AQP1; green), and DAPI (blue). Insets show higher-magnification views of the boxed region. Scale bars, 50 μm; inset, 25 μm. **b,** Ren1 mRNA expression across age (3, 6, 9, and 12 months) and sex. Data were analyzed by two-way ANOVA followed by Tukey’s post-hoc test (p=0.003; n = 4 – 8 animals per group as indicated). **c,** Representative images of choroid plexus explants isolated from the lateral ventricle (LV) and fourth ventricle (4V). Higher-magnification views of the boxed regions are shown below. Scale bars, 500 μm (low magnification) and 50 μm (high magnification). **d,** Representative western blots and quantification of renin protein expression in choroid plexus (ChP) and kidney (Kid) tissue from male and female mice. Recombinant mouse renin was included as a positive control. Two-way ANOVA followed by Uncorrected Fisher’s LSD revealed significant effects of sex (p=0.0266) and tissue type on renin protein abundance (p=0.0099). **e,f,** Representative coronal images of human choroid plexus showing REN mRNA (magenta) together with aquaporin 1 (AQP1; aqua) (**e**) or angiotensinogen (AGT; yellow) (**f**). DAPI is shown in blue. Insets show higher-magnification views of the boxed regions. Scale bars, 50μm; inset, 10μm.

Next, we sought to validate the RNA sequencing findings in both mouse and human tissue. Consistent with RNA-sequencing datasets, we identified *Ren1* mRNA expression in CPECs in mice (Fig. 2A). Analysis across the lifespan revealed a sex-specific pattern, with renin transcript levels peaking at 9 months in females and 6 months in males (Fig. 2B). We also assessed expression of other RAS members, including *Agt*, *Ace*, and *Ace2* (**Supp. Fig. 2A-C**). At the protein level, renin expression was detected in saline perfused-ChP explants (Fig. 2C). Strikingly, renin protein abundance in the ChP was comparable to that observed in the kidney of male mice; however, renin abundance was significantly less in the ChP compared to the kidney in female mice (Fig. 2D). Importantly, renin mRNA was also detected in human CPECs, where it exhibited some co-localization with angiotensinogen (Fig. 2E, F). These results confirm Ren1 or REN expression by mouse or human CPECs, respectively.

### ChP produces and secretes functional renin

**Figure 3.**
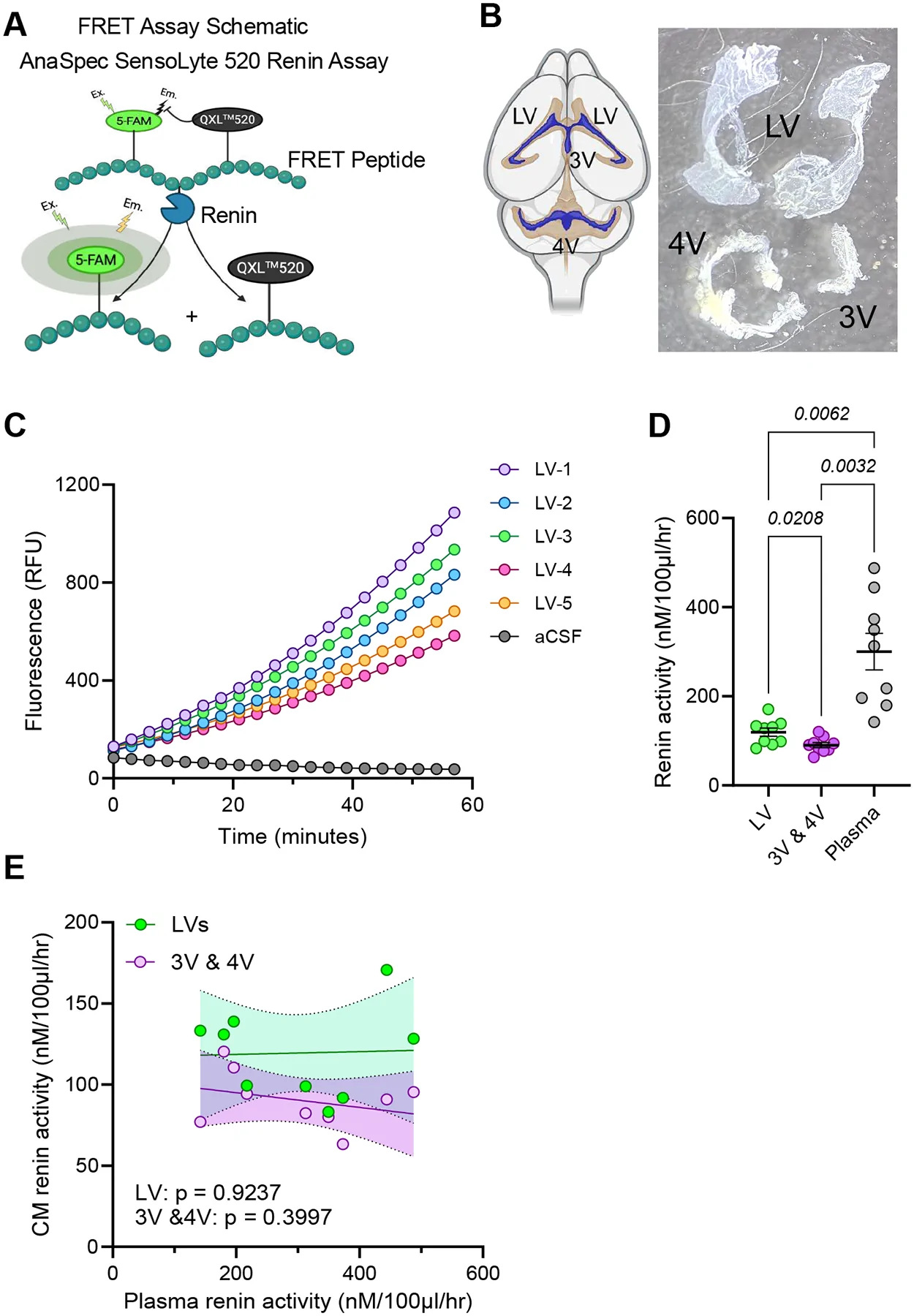
Mouse choroid plexus secretes functional renin. **a,** Schematic of the fluorescence resonance energy transfer (FRET)-based renin activity assay. Cleavage of the fluorogenic peptide substrate by renin separates the fluorophore from the quencher, resulting in increased fluorescence. **b,** Schematic illustrating the anatomical location of the choroid plexus within the mouse ventricular system and representative images of choroid plexus tissue following PBS perfusion and dissection. **c,** Renin activity in conditioned media from lateral ventricles over time (n=5 mice). **d,** Quantification of renin activity measured in conditioned media (CM) from pooled lateral ventricles, 3^rd^ and 4^th^ ventricles, and plasma (n=9 mice, paired one-way ANOVA with Tukey’s post-hoc test). **e,** Pearson r correlation analysis between ChP-secreted renin activity (nM/100μl/hr) into the CM and plasma renin activity (nM/100μl/hr) (n=9).

We next investigated whether the ChP can release renin and therefore is a source of functional renin. To this end, we used a fluorogenic mouse enzyme activity assay (Fig. 3A). This assay utilizes a quenched FRET peptide substrate containing a renin-specific cleavage site, which emits fluorescence upon enzymatic cleavage by active renin. We validated a lower limit of detection of 1.56ng/mL of renin and enzymatic activity could also be blocked by a renin inhibitor (**Supp. Fig. 3A-B**). To address this question, blood was collected for plasma isolation from mice prior to PBS perfusion. Following perfusion, ChP explants were isolated from all ventricular regions and incubated in artificial cerebrospinal fluid (aCSF) for 24 hours. Explants from the lateral ventricles were pooled, whereas explants from the third and fourth ventricles were combined (Fig. 3B). Renin activity analysis of conditioned media demonstrated that ChP explants secrete functional renin (Fig. 3C). Renin activity in conditioned media (CM) from ChP explants is lower than plasma renin activity, as would be expected (Fig. 3D). Interestingly, renin activity in CM from lateral ventricle explants was greater than that from 3^rd^ and 4^th^ ventricle explants, in agreement with Ren1 expression observed to be higher in the lateral ventricles in Fig. 1J. Moreover, ChP-derived renin activity was not correlated with plasma renin activity (Fig. 3D). These results indicate that the ChP is capable of producing and secreting functional renin.

### Renin activity in the CSF of humans

**Figure 4.**
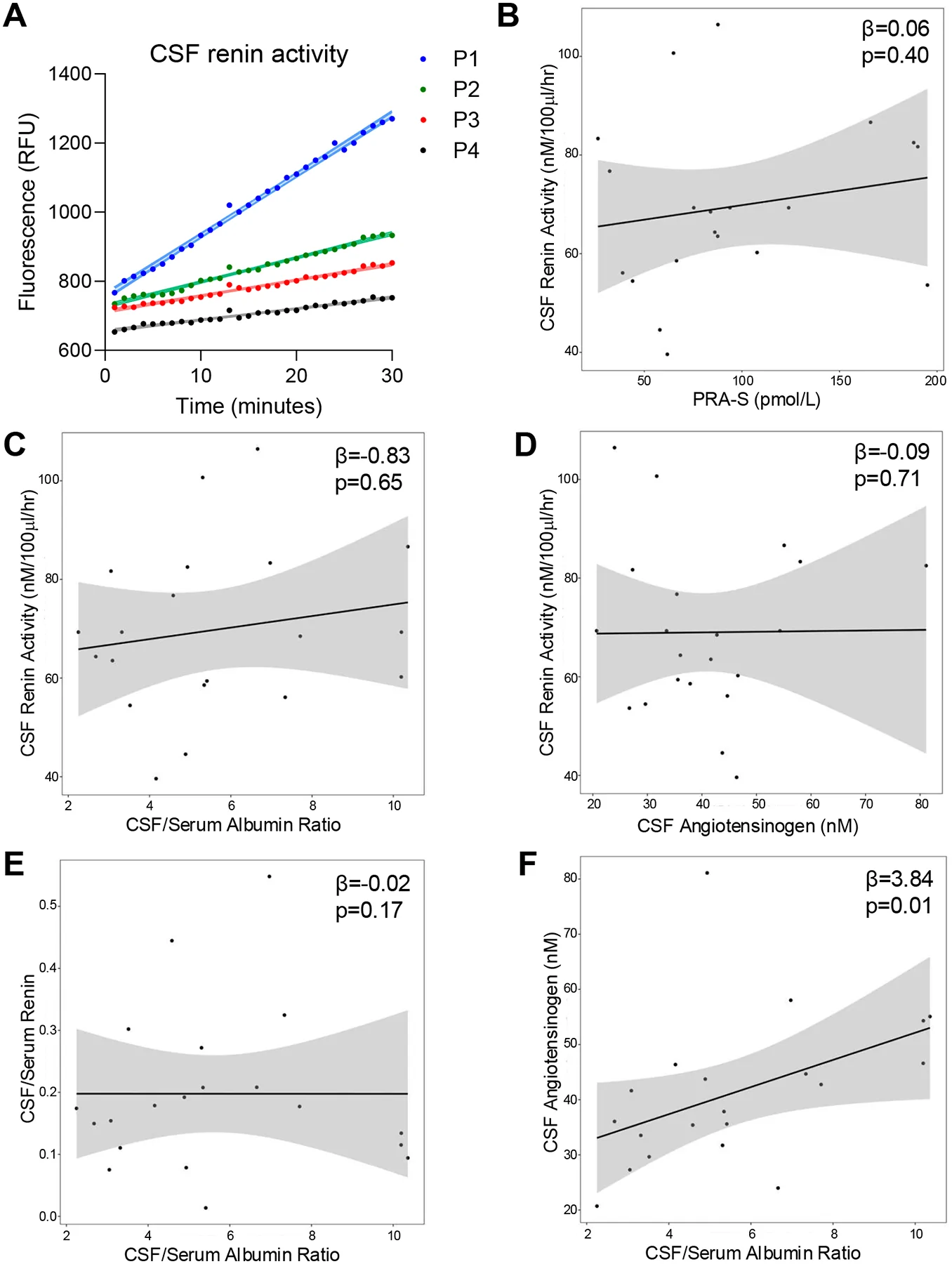
Renin activity is present in human CSF. **a,** The CSF renin activity from four participants (P1, P2, P3, P4) was measured and plotted over time. Linear regression models for **b,** CSF renin activity and plasma renin activity surrogate (PRA-S), **c,** CSF renin activity and CSF/Serum albumin ratio, **d,** CSF renin activity and CSF Angiotensinogen, **e,** CSF/Serum Renin and CSF/Serum albumin ratio, and **f,** CSF angiotensinogen and CSF/Serum albumin ratio. β is the effect estimate. The black line represents the fitted regression line, and the shaded area indicates the 95% confidence interval.

Next, we asked whether renin secreted into the CSF remains functional. To assess renin activity in human CSF, we employed a fluorogenic human enzyme activity. Like the mouse assay, the human assay can also be completely blocked by a renin inhibitor (**Supp. Fig. 3C**). First, CSF samples from four TAP participants were incubated with the FRET substrate, revealing measurable renin activity and demonstrating inter-individual variability in enzymatic activity (Fig. 4A). Notably, CSF renin activity from 20 participants **(Table 1)**, was not associated with circulating plasma renin (PRA-S) (Fig. 4B), CSF/serum albumin ratio (Fig. 4C), or CSF angiotensinogen levels (Fig. 4D). Similarly, the CSF/serum renin ratio did not correlate with CSF/serum albumin ratio (Fig. 4E), suggesting CSF levels are not derived from the periphery. In contrast, CSF angiotensinogen levels were significantly correlated with CSF/serum albumin (Fig. 4F), suggesting that angiotensinogen in the CSF may be impacted by brain barrier integrity. These results suggest that CSF renin is not affected by barrier integrity and is independent of circulating renin.

### Renin activity increases with age

**Figure 5.**
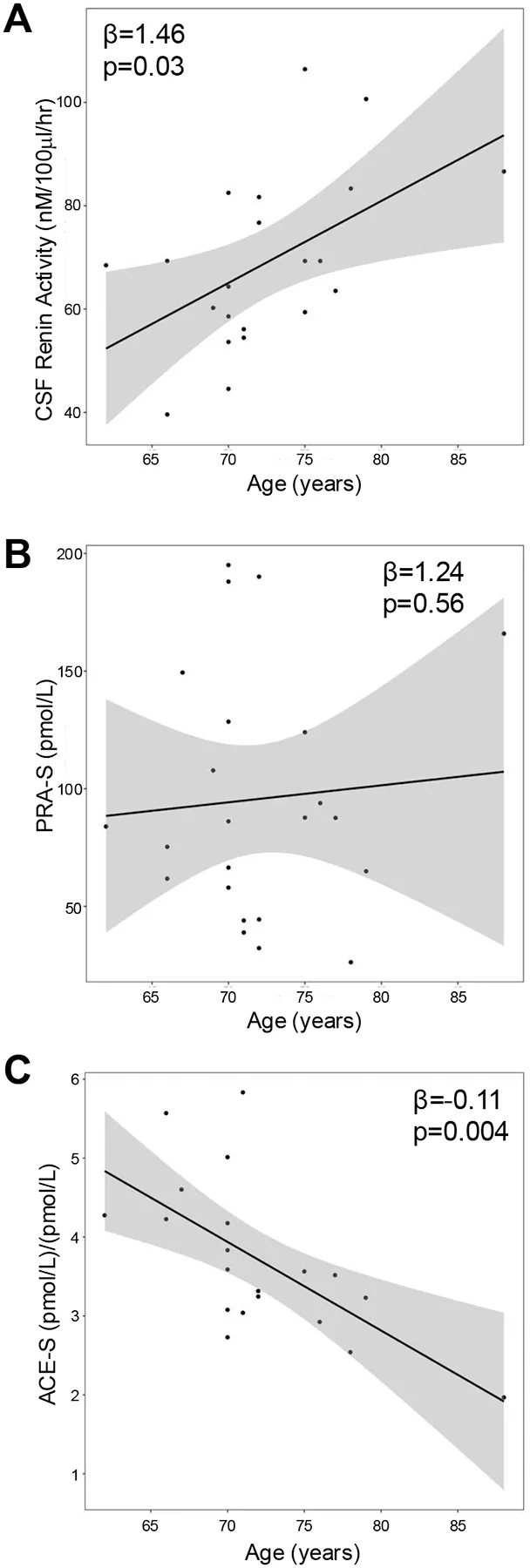
Aging increases renin activity in the CSF but not in the circulation. Linear regression models for **a,** CSF renin activity and Age, **b,** PRA-S and age, and **c,** ACE-S and age. β is the effect estimate. The black line represents the fitted regression line, and the shaded area indicates the 95% confidence interval.

Lastly, we assessed how RAS activity changes in both the circulation and CSF with aging. Older age was associated with higher levels of renin activity in the CSF (Fig. 5A), but was not associated with renin activity in serum (Fig. 5B). In contrast to renin activity, older age was associated with lower levels of ACE activity in serum (Fig. 5C). All other CSF and serum RAS components were not associated with age (**Supp. Table 2**). These results highlight the relevance of CSF renin, as well as the renin angiotensin system, to human physiology during aging.

## Discussion

Renin, the rate-limiting enzyme in the generation of angiotensin II (Ang II), was discovered in the 1890s and decades later reported to be functionally active in different tissues including the brain^1–4^. In 1971, Ganten and colleagues demonstrated the presence of a renin-like activity in the brain capable of generating angiotensin I independently of kidney and plasma renin^38^. Subsequently, Inagami and colleagues used anti-renin antibodies to map renin immunoreactivity throughout the brain, identifying signals in multiple regions of the central nervous system (CNS), including particularly high expression within the choroid plexus (ChP)^3^, also corroborated by a recent publication^12^. Crucially, purified mouse brain renin was demonstrated to be biologically active *in vivo*, as intracerebroventricular injection elicited changes in water intake and arterial blood pressure in rats^39^. Together, these studies suggest that the brain generates angiotensin peptides locally. Indeed, Ang I and Ang II were shown to be present in the brain of intact rats and in those that underwent bilateral nephrectomy as well as in collected CSF^40^.

On the other side of this argument, Schelling and colleagues failed to consistently measure renin activity in the CSF despite measurable levels of angiotensinogen and angiotensin-converting enzyme^41^. More recently, it has been argued that renin activity measured in the brain is trapped from the plasma and generated Ang II is not locally synthesized within the brain^42,43^. This controversy is not new. In 1970s, brain Ang I production was attributed to cathepsin D^44^, as Reid et al showed that brain renin has enzymological similarities to cathepsin D, a lysosomal acid protease^45^. The optimal enzymatic activity of cathepsin D is in the acidic range at pH 4 and very low activity at neutral pH of 7.4^46^. Subsequent studies^47–49^ showed that the actions of cathepsin D could be separated from brain renin, and only renin could generate Ang I at neutral physiological pH. Furthermore, anti-renin antibodies do not cross-react with cathepsin D^3^. Thus, the consensus in the field is that brain and CSF Ang I production is highly unlikely to be attributed to cathepsin D.

Despite this longstanding debate surrounding the existence of a functional brain RAS, largely driven by conflicting evidence regarding the presence and activity of renin within the CNS, substantial evidence supports its role in regulating blood pressure, metabolism, sympathetic and parasympathetic tone, and neuroendocrine outputs^8,50–52^. Particularly, brain specific renin-b has been established as a negative regulator of the central renin–angiotensin system, with studies demonstrating that loss of renin-b enhances sympathetic drive, elevates blood pressure, and disrupts fluid and metabolic homeostasis, thereby revealing a previously unrecognized mechanism controlling neurogenic cardiovascular regulation^5,6^. Importantly, dysregulation of the RAS has also been linked to cognitive decline and dementia^53–56^. Furthermore, treatment with RAS inhibitors is associated with a reduced risk of Alzheimer’s disease (AD) and slower cognitive decline, with greater benefits reported for those capable of crossing the blood–brain barrier^57,58^. Of note, neither renin nor Ang II cross the blood-brain barrier under healthy conditions. Thus, local production is necessary to mediate Ang II actions in the brain. Together, these observations suggest that understanding the source and regulation of renin in the central nervous system may have important implications for both cardiovascular and neurodegenerative diseases.

Recently, Fekete and colleagues provided compelling evidence supporting locally production of renin in the brainstem^12^. They identified robust expression of RAS components in a small population of cholinergic neurons in the nucleus ambiguus. Importantly, they demonstrated a functional role for these renin-expressing neurons in the regulation of autonomic balance. Conditional knockout of the renin-a isoform specifically in cholinergic neurons resulted in loss of renin expression in the nucleus ambiguus and led to alterations in autonomic function and baroreceptor reflex sensitivity in a sex-dependent manner. Interestingly, they also found renin mRNA expression in the ChP, corroborating RNA sequencing studies^17–21,23–35,59^ and further supporting previous evidence indicating its presence in the ChP^2,3,38,60^. While these findings establish a functional role for neuronal-derived renin in cardiovascular function, they do not address whether there is release of functional renin into the CSF for widespread actions throughout the brain. The ChP is particularly intriguing in this regard because it produces the majority of CSF and acts as a secretory organ releasing locally produced molecules into the CSF.

Here, we leveraged an interdisciplinary approach to provide evidence that the ChP is a source of functional renin within the central nervous system. By integrating transcriptomic, molecular, and functional approaches across mice and humans, we demonstrate that renin is expressed by CPECs, released into the CSF, and regulated independently of circulating renin, in agreement with prior studies^61^. These findings identify a previously unrecognized source of functional and widespread brain renin. Transcriptomic data indicates the presence of renin mRNA expression in CPECs, which we validated by in situ RNA hybridization (RNAscope), immunofluorescence, and western blotting. We found that the ChP has a comparable renin content compared to the kidney when compared per μg of tissue protein only in male mice. However, an important consideration is the kidney is notably larger than the ChP (in 3-month-old C57BL/6 mice, all choroid plexuses: <1 mg vs female kidney: 126.5±7.8 and male kidney: 178.4±9.4mg) and thus the absolute amount of renin produced by the kidney is exponentially higher than that produced by the ChP, as would be expected. Interestingly, we identified reduced ChP renin in young female mice compared to male mice, despite identical kidney renin, raising the possibility that central renin production is important for the partial protection against hypertension observed in female mice^62^.

The ChP is a highly vascularized epithelial structure located within the brain ventricles producing approximately 80% of CSF. Importantly, the ChP is a secretory organ capable of influencing brain function through CSF-mediated signaling^16^. Early work by Maktabi, Heistad, and Faraci found that the ChP is also a target of Ang II actions, as would be expected of a highly vascularized structure^63^. They found that both intravascular and intracerebroventricular Ang I and Ang II decreased blood flow to the ChP, despite not reducing cerebral blood flow, both measured by using microspheres^64^. The effect is mediated by Ang II, as ACE inhibitor administration prevented the decrease in blood flow in response to Ang I without affecting the response to Ang II. Along with a reduction in ChP blood flow, they found that Ang II also decreases the rate of CSF production^65^. Thus, it’s conceivable that local production of Ang II in the CSF could engage in a negative feedback loop to regulate ChP blood flow and CSF production, although this remains to be tested.

Here, we observed an association between older age and higher levels of CSF renin activity in human, but no age association with serum PRA-S. Since renin is the rate-limiting step in the renin angiotensin system, it crucially regulates Ang II production. The age-associated increase in CSF renin activity is important because Ang II promotes neuroinflammation^66,67^, neuronal stress^68^, amyloid beta production^69^, and Ang II is proposed to be important in dementia^70^. This is also relevant in the context of studies showing that ACE1 protein and enzyme activity are increased in the brains of individuals with AD compared to age-matched controls^71^. Future studies will investigate whether CSF renin activity is altered in individuals with AD and related dementias.

Additionally, understanding how renin in the ChP and CSF are regulated remains under investigation. Renin release in the kidney is regulated by three main mechanisms: changes in blood pressure, macula densa signaling in response to sodium depletion, and sympathetic nerve activation^72^. Previous studies found no correlation between CSF renin and blood pressure in humans^61^, thus raising the possibility that it is not modulated by blood pressure. Renin activity in the choroid plexus did not change during water deprivation in rats, although there appeared to be a trend towards a slight increase^60^. Additionally, it was increased by hypovolemia induced by subcutaneous polyethylene glycol, but decreased in nephrectomized rats after acute hemorrhage^73^, suggesting that the association with blood pressure and blood volume may be more complex. Further complicating our understanding of its regulation, acute unilateral or bilateral ligation of the internal carotid artery of rats led to a decrease in ChP renin, while chronic ligation resulted in an increase in ChP renin. Sodium may also be playing a role in regulating ChP and CSF renin, as CSF renin activity was increased with sodium depletion in dogs, although to a lesser extent than plasma renin activity (6.6 fold in plasma vs 1.3 fold in CSF)^74^. Lastly, the role of sympathetic nerves in modulating ChP and CSF renin remains unexplored. The ChP has extensive sympathetic innervation, and their activation leads to a decrease in CSF production by reducing ChP blood flow^75^. Thus, the regulation of renin released by the ChP into the CSF remains incompletely understood and must be resolved by ongoing investigation.

## Methods

### RNA sequencing analysis of publicly available datasets

Datasets were obtained from 18 published works^17–21,23–36^ and 1 unpublished work^22^ from three repository sources: NCBI geo, UCSC cell browser, and The Broad Institute single cell portal. For datasets that were available as processed single cell Seurat RDS file, the RDS file was downloaded and mined with standard Seurat gene expression workflow.

For single cell or nuclear datasets that were available as unprocessed filtered gene matrix, the matrix, barcode, and feature files were downloaded and put through the standard Seurat^76^ pipeline to yield a Seurat object that was subjected to gene expression mining. The raw counts for the bulk RNA seq datasets were downloaded and processed with DEseq2^77^ workflow. The metadata for datasets was downloaded along with the matrix files and the labels (cell identify, conditions, sex, age, experimental details) were applied. For all datasets, the first objective was to subset the CPECs and the cell ID annotation supplied by the authors of the studies were used to subset the cell type of interest.

Single cell/nuclear datasets^17,19–21,24,28,31,36^ were merged following the Seurat V5 integration pipeline^76^ separately mined for the gene expression of renin, angiotensin, and angiotensinogen. Feature and violin plots for the expression of the genes in all CPECs were generated along with correlation of expression across the three genes of interest. The renin expressing CPECs were then isolated and subjected to expression of the genes of interest along with Pearson correlation of expression of the genes.

To compare all datasets, both single- and bulk- seq datasets, the single cell/nuclear datasets were converted into a pseudo-bulk dataset following the standard ‘pseudo-bulk’ workflow provided by Seurat^76^. The pseudo-bulk and bulk studies were then merged, and all datasets were converted to the mouse nomenclature for the gene name using Ensembl’s BioMart package^37^. Standard DEseq2 pipeline was applied to normalize and correct for batch differences across the datasets. Normalized counts of the genes of interest were plotted across variables of interest and relative expression of the genes of interest were plotted as heatmaps.

### Animals

All procedures were approved by the Institutional Animal Use and Care Committee of Vanderbilt University Medical Center. Adult female and male C57BL/6J mice (JAX #664) were utilized for this study. Animals ranged from 3–12 months of age as detailed in each figure legend.

### Mouse choroid plexus RT-qPCR

Mouse choroid plexuses were isolated and immediately flash frozen until use. Choroid plexuses were placed into TRIzol reagent (Fisher Scientific, Cat. #15596018). RNA isolation was performed using the RNeasy Mini Kit from Qiagen (Cat# 74104) following the manufacturer’s protocol, including the recommended phase-separation and isopropanol precipitation steps. RNA concentration and purity were assessed using a NanoDrop spectrophotometer, and 500 ng of total RNA was reverse-transcribed using the iScript cDNA Synthesis Kit (Bio-Rad, Cat. #1708891) in a 20 μl reaction.

Quantitative PCR was performed using TaqMan Gene Expression Assays (Thermo Fisher Scientific) and TaqMan Gene Expression Master Mix on a QuantStudio 5 Real-Time PCR System. Mm-Ren1 (Mm02342887_mH), Mm-Agt (Mm00599662_m1), Mm-Ace (Mm00802048_m1), Mm-Ace2 (Mm01159006_m1), and Mm-Hprt (Mm03024075_m1) were used as probes (Thermo Fisher).

Reactions were run in 20 μl volumes in duplicate. Thermal cycling conditions followed the manufacturer’s recommendations (hold at 50 °C for 2 min, 95 °C for 10 min, followed by 40 cycles of 95 °C for 15 s and 60 °C for 1 min). Relative gene expression was calculated using the ΔΔCt method normalizing to Hprt and 3-month-old male group average.

### Western blot

Mice were anesthetized with isoflurane and transcardially perfused with 20 mL of ice-cold PBS. Following perfusion, the choroid plexus (from all the ventricles) and a defined cortical region of the kidney (approximately one-third of the organ) were dissected to assess renin protein content. Mouse renin recombinant enzyme (0.5 g, RayBiotech cat# 230-01252-50) was used as positive control. Immediately after isolation, tissues (n = 4 mice) were transferred to ice-cold Radioimmunoprecipitation Assay (RIPA) buffer (50 mM Tris-HCl, pH 8.0, 150 mM NaCl, 0.5% deoxycholic acid, 0.1% sodium dodecylsulfate (SDS), 1 mM EDTA, pH 8.0, 1% IGEPAL CA-630) supplemented with EDTA-free protease inhibitor (Millipore Sigma, Cat# 11836170001), 1mM sodium orthovanadate (Millipore Sigma, Cat #5086050004), and 20 mM sodium fluoride (Fisher Scientific, Cat #AAA1301930). Samples were homogenized by sonication, centrifuged (13,000 rpm for 10 min at 4°C), and supernatants were collected for downstream analysis.

Protein concentrations were quantified using the DC Protein Assay Kit II (Bio-Rad, Cat# 5000112). Lysates were denatured in Laemmli sample buffer containing dithiothreitol (DTT) at 100 °C for 10 min and resolved on Novex WedgeWell 4–12% Tris-Glycine 1.0-mm Mini protein gels (Thermo Fisher, Cat# XP04125BOX) for 80 min at 150 V. Proteins were transferred to polyvinylidene difluoride (PVDF) membranes (Thermo Fisher, Cat# 88518), which were then blocked for 1 h in 5% Bovine Serum Albumin (BSA) with 0.1% Tween-20 in PBS and incubated overnight at 4°C with Mouse Renin 1 antibody (R&D Systems, Cat# AF4277) followed by monoclonal anti-β-actin antibody (Millipore Sigma, Cat # A5441).

The following day, membranes were washed three times for 15 min in PBS containing 0.1% Tween-20 and incubated with mouse anti-goat IgG-HRP (Santa Cruz, Cat# sc-2354) or goat anti-mouse IgG (Jackson Immuno, Cat # 115-035-146) for 1 h at room temperature. Immunoreactive bands were visualized using the Clarity Western ECL Substrate (Bio-Rad, Cat# 1705061) on an iBright Imaging System (Invitrogen). Images were analyzed using Image Lab software (Biorad).

### Mouse choroid plexus explants immunofluorescence

PBS-perfused choroid plexus explants were post-fixed in 4% paraformaldehyde (PFA) overnight at 4°C and processed as free-floating sections. Samples were permeabilized in 0.5% Triton X-100 in PBS for 1 h at room temperature, followed by blocking in 10% normal donkey serum (NDS) in 0.1% Triton X-100/PBS. Sections were incubated overnight at 4°C with rabbit anti-renin antiserum (1:600, a generous gift from Prof. T Inagami, Vanderbilt University to Dr. Harris^78^) diluted in 5% NDS/0.1% Triton X-100/PBS. The following day, tissue was washed three times for 15 min each in 0.1% Triton X-100/PBS and then incubated with the appropriate fluorophore-conjugated secondary antibody for 2 h at room temperature in 2% NDS/0.1% Triton X-100/PBS.

### Confocal imaging acquisition

Human and mouse tissue sections were imaged using a Zeiss LSM 880 laser-scanning confocal microscope at the Vanderbilt Cell Imaging Shared Resource (supported by NIH grants CA68485, DK58404, and EY08126). Imaging parameters, including laser power, gain, and detector settings, were kept constant within each experimental series. Z-stack images were acquired at identical step intervals across samples and subsequently processed and analyzed using ImageJ (NIH).

### Human post-mortem choroid plexus

Anonymized formalin-fixed paraffin-embedded (FFPE) choroid plexus samples from post-mortem neurotypical human adult males (n=6) were obtained from the NIH NeuroBioBank (University of Miami, Miami, FL, USA). Average age at death for cases was 73 ± 8 years. The average postmortem interval from death to fixation of the choroid plexus was 25 ± 8 hours. The absence of neurological disease was determined by medical records, death certificates, and neuropathology reports. Tissue blocks were sectioned at 10 μm by the Translational Pathology Shared Resource core facility using a microtome and sections were stored at 4°C until processing. The Translational Pathology Shared Resource is supported by NCI/NIH Cancer Center Support Grant 2P30 CA068485–14.

### Multiplex Fluorescence RNAscope in-situ hybridization

Multiplex fluorescence RNAscope was performed on choroid plexus tissue according to the manufacturer’s instructions (Advanced Cell Diagnostics; Cat. #323280). Tissue preparation differed between human FFPE and mouse fresh-frozen samples, as described below. Following tissue preparation, sections were treated with hydrogen peroxide for 10 min at room temperature, subjected to target retrieval for 15 min at 100 °C, and incubated in 100% ethanol for 3 min. Slides were then air-dried overnight at room temperature. The following day, sections were incubated with Protease III for 30 min at 40 °C, followed by probe hybridization for 2 h at 40 °C. Signal amplification was performed using the RNAscope Multiplex Fluorescent v2 workflow. Positive and negative control probes were processed in parallel for both human and mouse samples.

### Human post-mortem tissue

For human post-mortem tissue, 10 μm FFPE lateral ventricle choroid plexus sections were baked at 60 °C for 1 h, deparaffinized in two changes of xylene, and rehydrated through two washes of 100% ethanol. After air drying, sections were processed using the RNAscope workflow described above. The following probes were used: Hs-REN (Cat. #401921) and Hs-AGT-C3 (Cat. #459131-C3; diluted 1:50 in C1 probe).

For dual detection of *REN* mRNA and aquaporin 1 (AQP1) protein, RNAscope was first performed using the Hs-REN probe. Sections were then blocked with 10% normal donkey serum (NDS) in PBS containing 0.1% Tween 20 for 1 h at room temperature, incubated with anti-AQP1 antibody (Abcam, Cat. #AB300463; 1:200) for 2 h at room temperature, washed three times in PBS containing 0.1% Tween 20 for 15 min each, and incubated with the appropriate fluorophore-conjugated secondary antibody for 1 h.

### Mouse fixed-frozen tissue

Mice were transcardiallly perfused with PBS followed by 4% paraformaldehyde (PFA). Brains were post fixed in 4% PFA followed by cryopreservation in 30% sucrose. Brain 10 μm sections containing the lateral ventricle choroid plexus were stored at −20°C until use, and were then allowed to dry overnight at room temperature. The next day, sections were baked, post-fixed in 4% PFA for 30 min at room temperature, and washed twice in PBS before RNAscope processing. The following probes were used: Mm-Ren1 (Cat. #433461) and Mm-Agt-C3 (Cat. #426941-C3).

For dual detection of *Ren1* mRNA and AQP1 protein in mouse tissue, RNAscope was first performed, followed by blocking with 10% NDS in PBS containing 0.1% Tween 20 for 1 h at room temperature. Sections were then incubated with anti-AQP1 antibody (Santa Cruz Biotechnology, Cat. #sc-32737) overnight, followed by incubation with the appropriate fluorophore-conjugated secondary antibody for 1 h the next day.

### Quantification of secreted renin from choroid plexus explants

Choroid plexus tissue was carefully dissected from all ventricles of PBS-perfused mice under a stereomicroscope. Explants, previously established to be viable in culture^79^, were immediately transferred into 125 μl oxygenated aCSF containing (in mM): KCl (2.5), NaCl (126), NaH_2_PO_4_ (1.2), CaCl_2_ (2.4), MgCl_2_ (1.2), NaHCO_3_ (25), glucose (11), L-ascorbic acid (0.4) and pH was adjusted to 7.4^80^. Explants were incubated at 37 °C in a humidified incubator maintained at 95% O_2_ and 5% CO_2_ for 24 h. Culture supernatants (conditioned media) containing released renin were collected and used for quantification of renin activity using a FRET-peptide-based assay.

### Renin activity by fluorogenic peptide substrate

Renin activity was measured from the CSF using fluorogenic peptide substrates (AnaSpec SensoLyte 520 Renin Assay, Cat# AS-72040 for human, Cat# AS-72161 for mouse) following manufacturer protocol. 50μl of human CSF or 50μl of mouse ChP explant conditioned media was incubated for 30 minutes at 37°C prior to addition of 50μl of renin 5-FAM/QXL^™^520 fluorescence resonance energy transfer (FRET) substrate containing the renin cleavage site. 5-FAM fluorescence, quenched by QXL^™^520, is released upon renin-mediated cleavage and was measured at 37°C (Promega GloMax) at 60-second intervals over 30 minutes for human CSF or 3-minute intervals over 60 minutes for mouse ChP conditioned media.

### Study cohort, serum collection, and lumbar puncture

The Tennessee Alzheimer’s Project (TAP) was launched in 2021 as the clinical cohort of the Vanderbilt Alzheimer’s Disease Research Center. To be included in this cohort, participants were required to speak English, be ≥55 years old, have a reliable study partner, and have adequate auditory and visual skills. At study entry, TAP participants were (a) cognitively unimpaired, (b) had mild cognitive impairment, or (c) had AD. To determine study eligibility, participants underwent medical record review, medical history review, and a clinical interview (including functional assessment and the Clinical Dementia Rating with a reliable informant). Exclusion criteria included magnetic resonance imaging (MRI) contraindication, history of major psychiatric illness, neurological disease (e.g., multiple sclerosis), head injury with loss of consciousness >5 minutes, and systemic illness that would affect follow-up participation. Lumbar puncture-specific exclusion criteria included use of blood thinners other than aspirin, history of bleeding disorder, or severe deformity of the lumbosacral spine. CSF and serum samples were selected from a subset of participants randomly selected for balance across sex, age, hypertensive status, *APOE* genotype, and cognitive diagnosis (n=21, 72.04±5.04 years old, 53% female, **Table 1**). The protocol, guided by the Belmont Report, was approved by the Vanderbilt University Medical Center Institutional Review Board, and written informed consent was obtained prior to data collection. Data, analytic methods, and study materials can be obtained by contacting the TAP principal investigator, Dr. Angela Jefferson.

Participants underwent a morning fasting venous blood draw, and samples were immediately stored at −80°C. Whole blood was centrifuged at 2000g and 4°C for 15 minutes, and serum was extracted and stored in ten 0.5mL aliquots.

Prior to the lumbar puncture procedure, participants were required to fast overnight but were encouraged to hydrate beforehand. The lumbar puncture was performed with the participant sitting unless clinical considerations necessitate placement in the lateral decubitus position. 25 mLs of CSF were collected with a polypropylene syringe using a Sprotte 25-gauge spinal needle (Teleflex Medical, Wayne, PA, USA) at the L2–3, L3–4, or L4–5 interspaces. Samples were immediately mixed and centrifuged, and supernatants were aliquoted in 0.5-mL polypropylene tubes and stored at −80°C.

### Human RAS components

CSF and serum RAS components were measured by ultra-pressure-liquid chromatography-tandem mass spectrometry by Attoquant Diagnostics^81^. In serum, aldosterone, Ang II and Ang I, were measured, and Aldosterone-to-Angiotensin II ratio (AA2), renin activity surrogate (PRA-S, calculated from the sum of Ang I and Ang II and validated against plasma renin activity^81^), and ACE2 activity were derived. In CSF, angiotensinogen and aldosterone were measured.

### Albumin quantification

Albumin levels (serum and CSF) were measured with the MesoScale R-Plex Human Album Assay (Cat# K1514VR-2) following the manufacturer Assay Protocol 1 in the R-PLEX Antibody Sets Singleplex Assays Insert. Serum samples were diluted 300,000-fold and CSF samples were diluted 5,000-fold in diluent 59 followed by diluent 100. Plates were read on an SQ 120 instrument. The calibration curve was fitted with a 4-parameter logistic (or sigmoidal dose-response) model with a 1/Y^2^ weighting. The albumin ratio was calculated as CSF albumin (mg/L) / plasma albumin (g/L).

### Statistical analysis

Statistical analysis for Fig. 2–3 and Supp. Fig. 2 was performed in GraphPad Prism 10. Data was tested for normality using the Shapiro-Wilk test. Unpaired, two-tailed Student’s t-test was performed to assess intergroup differences for two group comparisons. One-way or two-way analysis of variance (ANOVA) with Tukey’s multiple-comparison test or uncorrected Fisher’s Least Significant Difference were done, as appropriate.

### Human biospecimen analysis

Statistical analysis for Fig. 4–5, Supp. Table 2, and Supp. Fig. 4 was performed in R version 4.2.3. Nonparametric Wilcoxon rank-sum test was used to assess intergroup differences in Table 1. Spearman correlations were calculated to quantify unadjusted associations of interest, i.e. between independent variables (age, PRA-S, CSF angiotensinogen, and CSF/serum albumin ratio) and all outcome variables (CSF renin, serum aldosterone, CSF aldosterone, CSF/serum renin ratio, serum Ang II, PRA-S, ACE-S, and CSF angiotensinogen). We ran a series of linear regression models as detailed. First, we assessed the association of PRA-S, CSF/serum albumin ratio, and CSF angiotensinogen with CSF-renin adjusting for age and sex. Next, we assessed the association among CSF/Serum albumin ratio and CSF RAS measures (renin, angiotensinogen, and aldosterone) adjusting for age and sex. Lastly, we assessed the association among age and RAS measures (CSF and serum) adjusting for sex. Sensitivity analysis was performed to exclude outliers defined as values >4 standard deviations away from the group mean for all variables, and 1 outlier was excluded in PRA-S. For ACE-S analysis (Fig. 5C), n=4 participants were excluded for taking ACE inhibitors in their medications.

## Supplementary Material

Supplementary Files

This is a list of supplementary files associated with this preprint. Click to download.

- Table1.pdf
- Supplement.pdf

Table 1 is available in the Supplementary Files section.
